# Impact of grit, empathy, and communication competence on the clinical competence of nursing students in the post-COVID-19 era in Korea: a cross-sectional study

**DOI:** 10.7586/jkbns.24.041

**Published:** 2025-02-25

**Authors:** Soo-Hyun Nam, Seurk Park

**Affiliations:** Department of Nursing, Andong National University, Andong, Korea

**Keywords:** Students, nursing, COVID-19, Communication, Clinical competence, Empathy

## Abstract

**Purpose:**

This study investigated the relationships among grit, empathy, communication competence, and clinical competence in nursing students and aimed to determine the factors influencing their clinical competence during the coronavirus disease 2019 (COVID-19) pandemic.

**Methods:**

The participants were 157 third- and fourth-year nursing students from Andong National University in Andong and Choonhae College of Health Sciences in Ulsan, both in South Korea. Data were collected using a self-report questionnaire and analyzed using the SPSS version 23.0 program.

**Results:**

Clinical competence was positively correlated with grit (r = .26, *p* = .001), empathy (r = .29, *p* < .001) and communication competence (r = .53, *p* < .001). Women, sex (β = −.20, *p* = .009) was found to predict nursing students' clinical competence, explaining a portion of the variance in clinical competence (F = 7.07, *p* < .001).

**Conclusion:**

The results imply that nursing students’ communication competence must be increased to improve their clinical competence. Additionally, it is important to develop training programs that consider changes in the educational environment in the post-COVID-19 era.

## INTRODUCTION

The coronavirus disease 2019 (COVID-19) pandemic, initiated on March 11, 2020, has significantly impacted education globally, particularly in nursing [1,2]. Universities shifted to online learning and clinical practice was replaced with simulations, introducing nursing students to unprecedented learning environments [3-5].

The pandemic highlighted the need for innovative approaches to clinical training. Simulations and virtual reality (VR) emerged as powerful tools for creating realistic clinical environments, allowing students to develop skills and make clinical decisions in safe, controlled settings [3,5,6].

Communication competence plays a crucial role in enhancing nursing students' clinical competence, particularly in the post-COVID-19 era. The pandemic has emphasized the importance of effective communication competence in adapting to limited face-to-face interactions while maintaining quality patient care [6-8]. Research consistently shows that nursing students with higher communication competence exhibit improved clinical competence, enabling them to navigate complex scenarios more effectively and facilitate productive interactions with patients, caregivers, and medical staff [9-13]. These competencies also contribute to increased self-efficacy and adaptability, critical factors in responding to dynamic healthcare settings [14-16].

In response to the challenges posed by the pandemic, innovative educational methods such as simulation-based education, online role-playing, and VR have emerged as effective tools for strengthening communication competence [3,4,16-18]. These approaches allow students to practice essential skills in immersive and controlled settings, preparing them for the evolving landscape of nursing education and practice. As healthcare environments increasingly incorporate hybrid and technology-driven practices, integrating robust communication training into nursing education programs is essential to equip future nurses with the skills necessary to provide compassionate, effective care and meet the demands of modern healthcare [3,4,16].

Empathy remains a vital skill for nursing students, significantly contributing to building rapport and fostering effective communication with patients and healthcare teams [19,20]. Research highlights that empathic understanding enhances communication skills, academic self-efficacy, and clinical performance among nursing students [21,22]. Higher levels of empathy have been associated with better clinical competence, which is especially crucial during times of limited face-to-face interactions, such as the COVID-19 pandemic [6,8,9,13]. Even in online learning environments, educational interventions aimed at fostering empathy—such as simulation-based education, online role-playing, and virtual reality—positively impacted students' clinical competence [4,16,23,24].

In addition to empathy, nursing students must acquire professional knowledge and develop competencies to respond effectively to clinical situations. Grit—the perseverance and passion for achieving long-term objectives—plays a crucial role in overcoming challenges, maintaining motivation, and achieving success in nursing education and practice, particularly in the evolving post-pandemic healthcare environment [25-27]. By fostering both empathy and grit, nursing education programs can prepare students to navigate complex clinical scenarios with resilience and compassion, ensuring they deliver high-quality care in the face of adversity [2,4,21,22,25].

Grit has emerged as a crucial factor positively influencing nursing students' clinical competence, particularly in the context of the challenges presented by the COVID-19 pandemic [28]. Research indicates that nursing students with higher levels of grit demonstrate better clinical competence, even amid altered learning environments [25,29]. Grit enables students to overcome difficulties, persist toward long-term goals, and adapt to rapidly changing educational and clinical settings [26,27]. In the post-COVID-19 era, grit has been found to indirectly impact clinical competence through its influence on self-efficacy and adaptive coping strategies [24,30,31]. These traits have become even more essential as students navigate the uncertainties and challenges of a pandemic-affected educational landscape [14,30,32].

The COVID-19 pandemic has significantly impacted nursing students' clinical competence, disrupting traditional hands-on learning opportunities [4,24,33]. However, studies have indicated that grit, empathy, and communication skills can help mitigate the negative effects of these challenges [9,21,25,31]. Nursing students who demonstrate higher levels of grit are better able to cope with uncertainty and stress, while those with strong empathy and communication skills maintain higher levels of confidence in patient care, despite limited opportunities for in-person clinical training [15,21,25,34]. Research suggests that fostering these competencies may help mitigate the impact of the pandemic on nursing students' clinical competence, providing a foundation for more resilient and adaptable healthcare professionals in the post-pandemic era [25,28,31].

These findings underscore the importance of grit, empathy, and communication competence in supporting nursing students through pandemic-related challenges and enhancing their clinical competence in an increasingly complex healthcare environment. As nursing education faces a crossroads in the post-COVID-19 era, this study aims to explore the relationships between these attributes and clinical competence among nursing students. We can develop strategies to better prepare future nurses for the evolving healthcare landscape by analyzing these factors.

The findings of this study will contribute to the creation of educational approaches that align with the new realities of nursing practice and education in the post-pandemic world. More specifically, this study aimed to:

1) Investigate the level of grit, empathy, communication competence, and clinical competence among nursing students.

2) Identify differences in nursing students’ clinical competence based on their general characteristics, grit, empathy, and communication competence.

3) Determine the relationship between the grit, empathy, communication competence, and clinical competence of nursing students.

4) Identify the factors that influence the clinical competence of nursing students.

The results of this study can help develop strategies that enhance nursing students’ clinical competence in a changing educational environment.

## METHODS

### 1. Study design

This descriptive survey examined the relationship between grit, empathy, communication competence, and clinical competence among nursing students in the post-COVID-19 era. It also analyzed how grit, empathy, and communication competence affect nursing students’ clinical competence.

### 2. Participants

This study targeted third- and fourth-year nursing students enrolled in Andong National University in Andong City and Choonhae College of Health Sciences in Ulsan City, both in South Korea. The participants were students who understood the purpose of the study and provided written consent to participate. The number of participants was calculated using the G*power program 3.1.9.4 based on Cohen’s power analysis [35]. For the hierarchical regression analysis, the minimum required sample size was calculated to be 157. This calculation was based on an effect size of 0.15, a significance level of 0.05, a statistical power of 0.90, and 12 predictors (9 general characteristics, grit, communication competence, and clinical competence). After excluding three students who did not respond to the questionnaire, the sample size in this study was 157.

### 3. Instruments

#### 1) Grit

Grit was measured using the Grit Measurement Instrument developed by Duckworth and Quinn [36] and adapted to Korean by Song and Lim [37]. We verified this instrument for validity and reliability among Korean nursing students after obtaining the approval of the developers. The instrument comprises two subscales: maintaining interest (three items) and sustaining effort (three items). All items are rated on a 5-point Likert scale ranging from 1 (“not at all true”) to 5 (“very true”). Negative items are reverse-scored, and higher scores indicate higher levels of grit. The Cronbach’s α of the instrument ranged from .73 to .83 in Duckworth and Quinn’s [36] study. In Song and Lim’s [37] study, the Cronbach's α was .73, .77, and .69 for the entire instrument, the subscale of sustaining effort, and the subscale of maintaining interest, respectively. In this study, the Cronbach's α was .80, .73, and .84 for the entire instrument, the subscale of sustaining effort, and the subscale of maintaining interest, respectively.

#### 2) Empathy

Empathy was measured using the Interpersonal Reactivity Index developed by Davis [38] and adapted to Korean by Kang et al [39]. The index comprises 28 questions across four domains: perspective-taking, fantasy, empathic concern, and personal distress. The domains of perspective-taking and fantasy represent cognitive empathy subscales, while the domains of empathic concern and personal distress represent affective empathy subscales. Each domain comprises seven questions, and all questions are answered on a Likert scale ranging from 1 (“This sentence does not express me well”) to 5 (“This sentence expresses me very well”). Nine questions are reverse-scored, and higher scores indicate higher levels of empathy. In Kang et al.'s [39] study, the Cronbach's α values were .80 for the entire index, .81 for the domain of imagining, .61 for the domain of perspective-taking, .73 for the domain of empathic concern, and .71 for the domain of personal distress. In the current study, the Cronbach's α for the entire index was .90, with values of .71, .75, .64, and .81 for the respective domains.

#### 3) Communication competence

Communication competence was assessed using Yang and Hwang’s [40] Korean Health Communication Assessment Tool. This tool assesses the communication skills of nursing students through instructor observation. We modified the tool to fit the Korean context and verified it for validity and reliability after obtaining the approval of the developers. The tool consists of 15 items rated on a Likert scale ranging from 1 (“not at all”) to 5 (“very much”). Higher scores indicate better communication competence. The Cronbach’s α of the tool was .85 in Yang and Hwang’s [40] study and .87 in this study.

#### 4) Clinical competence

Clinical competence was measured using the instrument developed by Lee et al. [41] and modified and supplemented by Choi [42] based on Schwirian’s [43] six-dimension scale after obtaining the approval of the developers. The instrument comprises 45 items across five factors: nursing process (11 items), nursing skills (11 items), education/cooperation (8 items), interpersonal/communication (6 items), and professional development (9 items). All items are rated on a 5-point Likert scale ranging from 1 (“very poor”) to 5 (“very good”). The total score ranges from 45 to 225, and higher scores indicate better clinical competence. The Cronbach’s ⍺ of the instrument was .92 in Choi’s [42] study and .97 in this study.

#### 5) Academic performance

Academic performance was categorized based on the school's grade point average percentile conversion table as follows: "high" for scores of 90 points or above, "medium" for scores between 60 and 89 points, and "low" for scores below 60 points.

### 4. Data collection

This study was conducted among third- and fourth-year nursing students enrolled in Andong National University in Andong City and Choonhae College of Health Sciences in Ulsan City. Participants were those who understood the purpose of the study and gave written consent to participate in the study. Data were collected using a self-report questionnaire. We explained the purpose and procedures of the study, the risks and benefits, the confidentiality of participants’ data, and the right to withdraw from participation at any time. Data collection began after obtaining approval from the institutional review board, and questionnaires were distributed and collected until January 30, 2024.

### 5. Data analysis

The collected data were analyzed in the following manner. First, the general characteristics of the participants were analyzed using frequencies and percentages and grit, empathy, communication competence, and clinical competence were analyzed using means and standard deviations. Second, we investigated differences in participants’ clinical competence based on their general characteristics using t-tests and one-way analysis of variance (ANOVA). For ANOVA, post-hoc analyses were conducted using Scheffé's test to identify specific group differences. Third, the relationship between grit, empathy, communication competence, and clinical competence was analyzed using Pearson correlation coefficients. Finally, we identified the determinants of nursing students’ clinical competence. Variables related to clinical competence among the general characteristics were entered into the regression model in the first step to control their explanatory power. Then, grit, empathy, and communication competence were entered in the second step to conduct hierarchical multiple regression analysis. All statistical analyses were conducted using the SPSS version 23.0 program (IBM Corp., Armonk, NY, USA).

### 6. Ethical considerations

All procedures were approved by the Institutional Review Board of Andong National University (IRB No: 1040191-202310-HR-015-01). The purpose and methods of the study, research procedures, possibility of withdrawal from the study, how to fill out the questionnaire, and the time required were explained to the participants, and their written consent was obtained. We also explained that the collected data would be anonymized and used for research purposes only and that they could withdraw at any time during the study without any penalty. All personal data and materials related to the study were coded and stored in a secure cabinet such that they could not be recognized by anyone other than the researcher. After completing the questionnaire, the participants were given a small gift as a token of appreciation.

## RESULTS

### 1. General characteristics of the participants

A total of 157 nursing students participated in the study, with a mean age of 23.42 ± 5.02 years. The majority of participants were women (79.0%), in their fourth year (51.6%), and had medium academic performance (61.8%). Most participants were satisfied with their nursing major (59.9%), clinical practice (57.3%), and interpersonal relationships (68.8%). Regarding the importance of empathy, 40.2% of the participants said it was important, while 54.1% said that it was very important (Table 1).

### 2. Difference of clinical competence according to general characteristics

Clinical competence differed significantly based on gender (t = 2.12, *p* = .035), academic year (t = −2.55, *p* = .012), academic performance (F = 3.23, *p* = .042), satisfaction with one’s major (F = 4.93, *p* = .008), satisfaction with one’s clinical practice (F = 9.29, *p* < .001), and perceived importance of communication competence (F = 3.53, *p* = .032). More specifically, better clinical competence was associated with a higher academic year, better academic performance, greater satisfaction with one’s major, greater satisfaction with one’s clinical practice, and more favorable perceptions of the importance of communication competence (Table 1).

### 3. Levels of grit, empathy, communication competence, and clinical competence

The mean score for grit was 3.72 ± 0.74, and the mean score for maintaining interest (3.73 ± 0.69) was higher than the mean score for sustaining effort (3.54 ± 0.92). The mean score was 3.62 ± 0.91 for empathy, 3.76 ± 0.76 for communication competence, and 3.90 ± 0.66 for clinical competence (Table 2).

### 4. Correlation between grit, empathy, communication competence, and clinical competence

Clinical competence was significantly and positively correlated with grit (r = .26, *p* = .001), empathy (r = .29, *p* < .001), and communication competence (r = .53, *p* < .001). More specifically, better clinical competence was associated with higher levels of grit, more empathy, and better communication competence (Table 3).

### 5. Factors influencing clinical competence

The assumptions for the hierarchical multiple regression analysis were met. The correlation coefficients between independent variables ranged from .19 to .53 (i.e., they did not exceed .80), indicating that there were no concerns of multicollinearity. The variance inflation factor ranged from 1.13 to 4.86, and tolerance ranged from 0.07 to 0.83, further confirming the absence of multicollinearity. The Durbin-Watson statistic was 1.79 (i.e., close to 2), indicating no autocorrelation of the residuals.

In Step I of the hierarchical regression, the variables that showed significant associations with clinical competence in the univariate analyses (sex, academic year, academic performance, satisfaction with one’s major, satisfaction with one’s clinical practice, and perceived importance of communication competence) were entered as predictors. This model was statistically significant (F = 3.90, *p* < .001), explaining 21.1% of the variance in clinical competence. The strongest predictor of clinical competence was being a women (β = −.20, *p* = .009).

In Step II, grit, empathy, and communication competence were added to the model. The final model was statistically significant (F = 7.07, *p* < .001), explaining 39.1% of the variance in clinical competence and showing an increase of 18.0% from Step I. Communication competence emerged as the strongest predictor of clinical competence (β = .41, *p* < .001), with better communication competence being associated with better clinical competence (Table 4).

## DISCUSSION

This study analyzed the factors influencing nursing students' clinical competence in the post-COVID-19 era. The results showed that clinical competence significantly differed based on sex, academic year, academic performance, satisfaction with one’s major and clinical practice, and perceived importance of communication competence. Furthermore, grit, empathy, and communication competence were positively correlated with clinical competence, with communication competence emerging as the strongest predictor.

The mean score for grit was 3.72 out of 5. This score is higher than the score reported in a previous study (3.30; [17]). The difference in the scores may be related to the academic year of the participants in the two studies. This study surveyed third- and fourth-year students, but participants’ academic year was not clearly specified in the other study. As students advance to higher academic years, their clinical practice and adaptation abilities tend to improve, and this can impact their grit scores.

Among the subscales of grit, the mean score for maintaining interest (3.73) was higher than the mean score for sustaining effort (3.54). These scores were higher than those reported in previous studies (maintaining interest: 2.76 [32] and 3.09 [25]; sustaining effort: 3.28 [19] and 3.34 [32]). Maintaining interest or sustaining efforts requires not just hard work but also long-term persistence and passion despite frustrations, failures, and adversities. However, during the COVID-19 pandemic, nursing students experienced various emotional changes owing to sudden shifts in teaching methods, difficulties in clinical practice, and increased pressure related to practicums. In this situation, educators strived to help students adapt by using different teaching methods, such as problem-based learning and flipped learning, and simulation-based education. These educational approaches may have helped nursing students maintain their interest and continue their efforts in the post-COVID-19 era.

The mean score for empathy was 3.62 out of 5. This score is similar to the score reported in a previous study involving students of the same academic year (3.43). These findings indicate that nursing students’ empathy levels are above average. Empathy plays a crucial role in how nurses respond to patients’ needs and form relationships, which, in turn, affect patient well-being and care quality [27,44]. Previous studies have shown that nurses with high levels of empathy provide patient-centered care based on a deep understanding of their patients and can effectively resolve interpersonal conflicts [8,27,32]. Research has also indicated that as the level of empathic concern improves, clinical competence also increases [8]. This suggests that empathy is closely related not only to forming relationships with patients but also to actual clinical practice skills. Therefore, further research is required to develop and validate educational programs that enhance nursing students’ empathy.

The mean score for communication competence was 3.76 out of 5. This score is lower than the score reported in a previous study involving third- and fourth-year nursing students (3.81) [28,29]. The difference in the scores may be attributed to the limited opportunities for practicing communication competence and nursing techniques in real clinical settings due to the COVID-19 pandemic compared to the period before the pandemic when normal clinical practice was possible [4,9]. In nursing education, simulation-based education has been suggested as an effective way to overcome the gap between theory and practice and enhance adaptability in clinical settings [17,37]. Therefore, it is necessary to develop and evaluate communication education programs for COVID-19 and future pandemics. These programs should include training on the use of personal protective equipment, video and telephone consultations, and case studies on communicating with patients and their families during a pandemic.

The mean score for clinical competence was 3.90 out of 5. This score is similar to or slightly lower than the scores reported in previous studies involving nursing students in the same academic year (3.49 and 3.72) [44,45]. This suggests that clinical experiences with actual patients decreased due to enhanced infection control measures and changes in clinical settings. Although simulations and online practices were used to complement the limited clinical practice, they may not have fully replaced nursing experiences in actual clinical environments. Therefore, it is necessary to develop and apply a hybrid practical training model that combines face-to-face practice with simulations using virtual or augmented reality technology.

Clinical competence differed significantly based on sex, academic year, academic performance, satisfaction with one’s major, satisfaction with one’s clinical practice, and perceived importance of communication skills. These findings align with those of previous studies showing that clinical competence tends to be higher among students who are women, those in higher academic years, those with higher academic performance, those with greater satisfaction with their major, those with greater satisfaction with their clinical practice, and those who consider communication skills important [9,14-18]. Students with high academic performance generally show a strong enthusiasm for task completion and have good study habits, which seem to translate into a high enthusiasm for clinical performance. Furthermore, students with high satisfaction with their major showed higher clinical competence and satisfaction with their clinical practice. To enhance nursing students’ satisfaction with their major, instructors should provide individualized guidance and counseling and implement extracurricular programs, such as major-related study groups, club activities, and mentoring programs.

Clinical competence was significantly and positively correlated with grit, empathy, and communication competence among nursing students. More specifically, better clinical competence was associated with higher levels of grit, more empathy, and better communication competence. These findings are consistent with the findings of studies that reported correlations between grit and clinical competence and relationships between empathy, communication skills, and clinical competence [20,25,28,29]. Therefore, it is necessary to develop programs that cultivate the grit, empathy, and communication competence of nursing students.

Communication competence was found to be the most significant predictor of clinical competence among nursing students, with the model explaining 39.1% of the variance in clinical competence. This finding aligns with the finding of previous studies [9,11,13] emphasizing the crucial role of communication skills in improving nursing students’ clinical competence. These findings underscore the importance of enhancing communication skills to improve clinical competence in nursing education. Therefore, nursing education programs should incorporate diverse educational strategies to improve communication skills. For instance, they can use role-playing exercises to reflect different clinical scenarios. They can also teach non-verbal communication techniques and analyze personal communication styles through video recordings.

The finding that grit and empathy did not significantly influence clinical competence in this study is noteworthy. This result contrasts with previous studies and may be related to changes in nursing education environments due to the COVID-19 pandemic.

The lack of significant impact of grit on clinical competence may be associated with changes in clinical practice environments due to COVID-19 [4,6]. Limited clinical practice opportunities and altered educational settings may have reduced students' chances to demonstrate grit in actual clinical situations [8,24]. Duckworth et al. viewed grit as a characteristic that emerges in long-term goal achievement processes; from this perspective, grit's influence may not have been clearly evident in a short-term changed environment [26,27].

The finding that empathy did not significantly affect clinical competence may be related to the shift from face-to-face to online learning during the pandemic [2,4]. Empathy skills, traditionally fostered through Human Relations and Communication courses, may have had limited opportunities for practice and development in real situations due to the predominance of video lectures and Internet platforms [14,15]. It also highlights the need for innovative educational approaches to cultivate empathy skills effectively within blended learning environments.

These results indicate the need to reconsider methods for effectively cultivating and evaluating grit and empathy in the changed nursing education environment post-COVID-19. Future research should include longitudinal studies spanning pre- and post-pandemic periods to observe changes in the influence of these factors, as well as qualitative studies exploring how grit and empathy manifest in changing clinical practice environments [21,22,28]. Such research efforts will help clarify the role and importance of grit and empathy in the evolving nursing education landscape.

One limitation of this study is that caution should be exercised in generalizing its results because the sample comprised nursing students recruited from specific regions. Nonetheless, this study is significant because it elucidated the relationships among grit, empathy, communication competence, and clinical competence and confirmed the necessity of enhancing these aspects to improve nursing students’ clinical competence. The findings of this study can serve as evidence for supplementing educational content to strengthen grit, empathy, and communication skills in nursing education and, consequently, improve nursing students’ clinical competence.

## CONCLUSION

By examining the effects of grit, empathy, and communication competence on the clinical competence of nursing students, this study aimed to provide foundational data for developing programs that enhance clinical competence. Communication competence was found to be the primary determinant of nursing students’ clinical performance, explaining 39.1% of the variance in clinical competence. The findings of this study can aid the development of educational strategies that increase nursing students’ clinical competence.

More specifically, we propose the following suggestions based on our results.

1) Further research is needed to examine the factors influencing nursing students’ grit and empathy, how these traits change across academic years, and their long-term impact on clinical competence.

2) To enhance nursing students’ communication skills, we recommend developing educational curricula and intervention programs that are tailored to the characteristics of nursing students.

3) Follow-up studies should be conducted to evaluate the effectiveness of programs aimed at improving nursing students’ communication skills.

4) To generalize the research findings, we suggest replicating this study with a larger sample size and geographical scope.

These endeavors can advance nursing education and improve nursing students’ clinical competence in the evolving healthcare landscape.

This study analyzes the impact of nursing students' grit, empathy, and communication skills on clinical performance, thereby providing new foundational knowledge necessary for nursing education post-COVID-19 and suggesting new research directions in fundamental nursing. Additionally, it presents educational strategies for training nurses who can adapt to the changing healthcare environment.

## Figures and Tables

**Table 1.** General Characteristics and Corresponding Differences in Clinical Competence (N = 157)

Variables	Categories	n (%)	Clinical competence		
			M ± SD	t or F	*p* (Scheffé test)
Age (yr)			23.42 ± 5.02		
Sex	Men	33 (21.0)	4.15 ± 0.79	2.12	.035
	Women	124 (79.0)	3.83 ± 0.61		
Academic year	3^rd^	76 (48.4)	3.79 ± 0.59	₋2.55	.012
	4^th^	81 (51.6)	4.00 ± 0.69		
Academic performance	High^a^	36 (22.9)	4.16 ± 0.74	3.23	.042 (a > b,c)^†^
	Medium^b^	97 (61.8)	3.91 ± 0.62		
	Low^c^	24 (15.3)	3.63 ± 0.61		
Satisfaction with nursing major	Satisfied^a^	94 (59.9)	4.03 ± 0.63	4.93	.008 (a > b,c)^‡^
	Neutral^b^	54 (34.4)	3.69 ± 0.64		
	Dissatisfied^c^	9 (5.7)	3.83 ± 0.75		
Satisfaction with clinical practice	Satisfied^a^	90 (57.3)	4.06 ± 0.65	9.29	< .001 (c > a,b)^§^
	Neutral^b^	57 (36.3)	3.61 ± 0.54		
	Dissatisfied^c^	10 (6.4)	4.10 ± 0.81		
Satisfaction with interpersonal relationships	Satisfied	108 (68.8)	3.94 ± 0.67	0.99	.375
	Neutral	43 (27.4)	3.85 ± 0.62		
	Dissatisfied	6 (3.8)	3.58 ± 0.66		
Importance of empathy	Common	9 (5.7)	3.92 ± 0.68	1.30	.274
	Important	63 (40.2)	3.92 ± 0.62		
	Very important	85 (54.1)	3.56 ± 0.68		
Importance of communication competence	Not particularly important^a^	2 (1.3)	3.00 ± 0.00	3.53	.032 (c > a,b)^∥^
	Important^b^	31 (19.7)	3.73 ± 0.51		
	Very important^c^	124 (79.0)	3.96 ± 0.68		

M = Mean; SD = Standard deviation.

† The high-performing group (a) scored significantly higher than both the medium (b) and low (c) performing groups;

‡ The satisfied group (a) scored significantly higher than both the neutral (b) and dissatisfied (c) groups;

§ Unexpectedly, the dissatisfied group © scored significantly higher than both the satisfied (a) and neutral (b) groups;

∥ The “very important” group (c) scored significantly higher than both the “not particularly important” (a) and “important” (b) groups.

**Table 2.** Grit, Empathy, Communication Competence, and Clinical Competence (N = 157)

Variables	Categories	M ± SD	Min ~ Max (range)
Grit	Maintaining interest	3.73 ± 0.69	2 ~ 5 (1 ~ 5)
	Sustaining effort	3.54 ± 0.92	1 ~ 5 (1 ~ 5)
	Total	3.72 ± 0.74	1 ~ 5 (1 ~ 5)
Empathy		3.62 ± 0.91	2 ~ 5 (1 ~ 5)
Communication competence		3.76 ± 0.76	1 ~ 5 (1 ~ 5)
Clinical competence		3.90 ± 0.66	2 ~ 5 (1 ~ 5)

M = Mean; SD = Standard deviation; Min = Minimum; Max = Maximum.

**Table 3.** Correlations among Grit, Empathy, Communication Competence, and Clinical Competence (N = 157)

Variables	Grit	Empathy	Communication competence	Clinical competence
	r (*p*)			
Grit	1	.25 (.002)	.19 (.016)	.26 (.001)
Empathy	.25 (.002)	1	.30 (< .001)	.29 (< .001)
Communication competence	.19 (.016)	.30 (< .001)	1	.53 (< .001)
Clinical competence	.26 (.001)	.29 (< .001)	.53 (< .001)	1
				

**Table 4.** Factors Influencing Clinical Competence (N = 157)

Variables	Categories	Model 1					Model 2				
		B	SE	ß	t	*p*	B	SE	ß	t	*p*
(Constant)		3.39	.52		6.49	< .001	1.29	.59		2.18	.031
Sex^†^	Women	−0.33	.12	−.20	−2.65	.009	−0.17	.11	−.11	−1.55	.123
Academic year^†^	4^th^	0.18	.10	.14	1.77	.080	0.02	.10	.02	0.21	.836
Academic performance^†^	High	0.27	.18	.18	1.53	.128	0.22	.16	.14	1.35	.179
	Medium	0.28	.15	.21	1.90	.059	0.18	.13	.13	1.32	.190
Importance of communication competence^†^	Important	0.45	.45	.27	1.00	.317	0.60	.40	.36	1.49	.139
	Very important	0.58	.44	.36	1.30	.197	0.65	.39	.41	1.65	.100
Satisfaction with nursing major^†^	Neutral	0.11	.27	.08	0.42	.678	−0.05	.25	−.04	−0.21	.835
	Satisfied	0.18	.28	.13	0.65	.519	−0.16	.25	−.12	−0.62	.537
Satisfaction with clinical practice^†^	Neutral	−0.46	.27	−.34	−1.74	.085	−0.08	.25	−.06	−0.34	.735
	Satisfied	−0.15	.27	−.12	−0.56	.575	0.21	.25	.16	0.85	.398
Grit							0.07	.06	.08	1.12	.265
Empathy							0.10	.06	.14	1.76	.081
Communication competence							0.36	.07	.41	5.51	< .001
F(*p*)		3.90 (< .001)					7.07 (< .001)				
R²		.21					.39				
adj R²		.16					.34				

SE = Standard error.

† Dummy variable: Sex (reference group: men); Academic year (reference group: 3^rd^); Academic performance (reference group: low); Importance of communication competence (reference group: common); Satisfaction with nursing major and Satisfaction with clinical practice (reference group: dissatisfied).

## Data Availability

Please contact the corresponding author for data availability.
